# Closed-loop functional optogenetic stimulation

**DOI:** 10.1038/s41467-018-07721-w

**Published:** 2018-12-13

**Authors:** Shriya S. Srinivasan, Benjamin E. Maimon, Maurizio Diaz, Hyungeun Song, Hugh M. Herr

**Affiliations:** 1 MIT Media Lab, Center for Extreme Bionics, Massachusetts Institute of Technology, Cambridge, 02139 MA USA; 2 Harvard-MIT Division of Health Sciences and Technology (HST), Massachusetts Institute of Technology, Cambridge, 02139 MA USA; 3 Department of Electrical Engineering and Computer Science, Massachusetts Institute of Technology, Cambridge, 02139 MA USA

## Abstract

Optogenetics has been used to orchestrate temporal- and tissue-specific control of neural tissues and offers a wealth of unique advantages for neuromuscular control. Here, we establish a closed-loop functional optogenetic stimulation (CL-FOS) system to control ankle joint position in murine models. Using the measurement of either joint angle or fascicle length as a feedback signal, we compare the controllability of CL-FOS to closed-loop functional electrical stimulation (CL-FES) and demonstrate significantly greater accuracy, lower rise times and lower overshoot percentages. We demonstrate orderly recruitment of motor units and reduced fatigue when performing cyclical movements with CL-FOS compared with CL-FES. We develop and investigate a 3-phase, photo-kinetic model to elucidate the underlying mechanisms for temporal variations in optogenetically activated neuromusculature during closed-loop control experiments. Methods and insights from this study lay the groundwork for the development of closed-loop optogenetic neuromuscular stimulation therapies and devices for peripheral limb control.

## Abstract (web summary)

Optogenetics is a promising alternative approach for restoration of neuromuscular function. Here the authors establish a closed-loop functional optogenetic stimulation for the control of limb joint angle in murine models, which demonstrates improved control and less fatigue than electrical stimulation systems.

## Introduction

Functional electrical stimulation (FES) is the current clinical standard used for stimulation to restore function and provide therapy in a variety of clinical applications. FES of nerve has been used to modulate respiration, bowel, bladder, and sexual function in spinal cord injury patients^1^. FES of muscle has been performed to improve muscle conditioning in patients with muscular degenerative diseases to aid walking and/or upper extremity function^2–4^. In all cases, electrical stimulation performs reverse order recruitment, activating large motor units (large-diameter axons innervating large, fast-twitch, oxidative, and glycolytic muscle fibers) before small motor units (small-diameter axons innervating slow, fatigue-resistant muscle fibers)^5–7^. Given this order, FES tends to provide coarse motor control, predominantly allowing access to only high levels of force output from a muscle. Small, slow twitch fibers, which provide lower magnitudes of force, cannot be selectively recruited, leading to quick fatigue^7^. When FES is applied transdermally, cutaneous mechanoreceptors, nociceptive fibers, and non-target muscles are often co-activated, limiting specificity and eliciting painful sensations^8^.

Recent advances in optogenetics, including transdermal approaches^9^ and tissue-selective induction^10^, have enabled optogenetic stimulation for control applications in the peripheral nervous system. Optogenetic stimulation allows biochemical neuromodulation, provides cell-type specificity, and is time-resolved to the millisecond timescale^11^. With the current open-loop optogenetic stimulation method, muscles demonstrate increased performance and reduced fatigue as compared to FES of the same nerve^5^.

Despite the body of work performed in the peripheral nervous system, no closed-loop (CL) studies utilizing optogenetics for motor control have been carried out. Commonly used to control paralyzed^2,12,13^ and prosthetic limbs^14,15^, CL systems provide greater functionality and practicality for users compared to their open-loop counterparts^16^.

Here, we establish a closed-loop functional optogenetic stimulation (CL-FOS) system for the control of joint angle in both rat and mouse models. We perform optogenetic stimulation of either the peroneal or tibial nerves, eliciting movement of the ankle joint. Ankle position or fascicle state was measured and fed into a closed-loop PI controller, which modulated the stimulation to bring the limb to the desired joint angle. Both non-invasive and invasive approaches were taken to validate the architecture using joint angle (non-invasive) and direct fascicle state (invasive) sensing. Using various control tasks, we elucidate the properties of CL-FOS and assess its fitness as a functional modality for neuromuscular applications. We test an equivalent closed-loop system utilizing electrical stimulation (CL-FES) and compare it to the CL-FOS system.

We hypothesize that transdermal CL-FOS is superior to CL-FES of nerve for joint angle control and cyclical use.

We evaluate the hypothesis using standard metrics such as rise time, steady-state error, and time constants of decay and fatigue. We further hypothesize that CL-FOS will induce less fatigue than CL-FES when performing cyclic movements. Our experimentation yields patterns in system behavior that inform us about the orderly recruitment mechanism of FOS.

Across our experimentation in various murine models, optogenetic expression levels, control signal waveforms, levels of stimulation invasiveness and two hind limb nerves, we observe a nonlinear and time-variant system response under CL-FOS. To characterize this temporal system response and modulation of optogenetic nerve excitability, we develop and investigate a 3-phase, photo-kinetic model, which consists of (1) activation, (2) inactivation, and (3) reactivation. We provide evidence to support this model and its key mechanistic determinants. This model lays the foundation for designing efficient optogenetic control systems and has significant implications for understanding the fatigue of biological systems under chronic optogenetic control.

## Results

### Design and performance of closed-loop system

All CL-FOS experiments were performed on mice expressing ChR2 transgenically (*n* = 10) or rats expressing ChR2 through viral transduction (*n* = 12). All CL-FES experiments were performed in rats (*n*=11). We confirmed the robust expression of ChR2 in virally transduced rats and transgenic mice through immunofluorescence of harvested spinal cord and nerve tissues. Expression was found to be uniform and present along the length of the nerve and in lumbar ventral horn neurons in the spinal cord (Supplementary Figure 2).

**Fig. 1:**
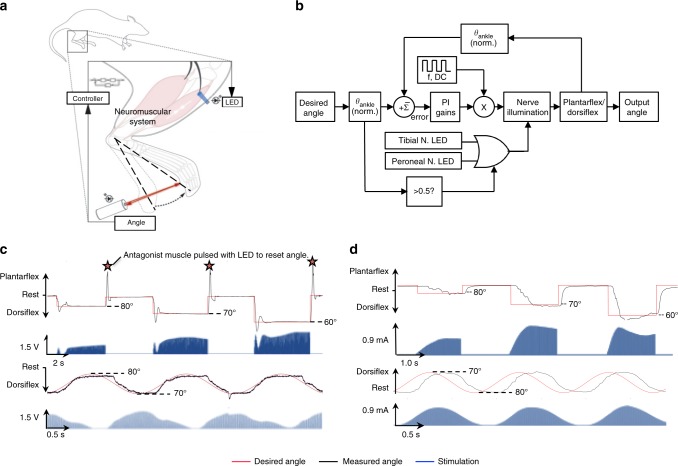
Closed-loop optogenetic system. **a** Schematic of system design for CL-FOS. The peroneal or tibial nerve was optically stimulated using an LED, resulting in a contraction of the tibialis anterior or gastrocnemius muscle, yielding dorsiflexion or plantarflexion, respectively. Measurement of the footpad displacement was fed into the controller, which modulated the stimulation intensity to bring the limb to the desired angle. **b** Control diagram depicting pseudo-SITO (single input, two outputs) structure employed for CL-FOS control of the joint angle with PI gains where f: frequency, DC: duty cycle. **c** Representative trial of CL-FOS accuracy under PI control utilizing a virally transduced rat. Average SSRMSE was 3.01 ± 1.32% for square wave patterns and 1.16 ± 0.93% for sinusoidal patterns (*n* = 12). **d** Representative trial of CL-FES accuracy under PI control utilizing a virally transduced rat. Average SSRMSE was 6.31 ± 2.98% for square wave patterns and 11.18 ± 4.32% for sinusoidal patterns (*n* = 11)

The CL-FOS and CL-FES systems comprised of a PI controller (Fig. 1a, b), which modulated optical illumination or electrical stimulation of hind limb nerves to drive the joint angle through control patterns. The actual position of the ankle was measured by a distance sensor and fed to the controller to determine the error between the desired and measured positions. FOS was delivered at 1.25 ms pulse width (PW) and 40 Hz frequency to induce tetanizing contraction, while minimizing fatigue and limiting heating of the light source. For FES, an array of PWs was tested to determine the sensitivity of the system to the FES PW. We determined no significant changes to system response for pulse widths over the range of 0.10 ms to 1.60 ms (Supplementary Figure 3) and thereby delivered pulses conservatively at 0.10 ms to minimize fatigue. Optimal gains for the controller were determined for each animal by analyzing the stability of movement, and minimizing ringing and overshoot. We have described this process in greater detail and provided representative data in Supplementary Figure 4.

The optimized system was subjected to square and sinusoidal control waveforms under CL-FOS (*n* = 12) and CL-FES (*n* = 11) (Fig. 1c, d). Control properties, including accuracy, rise time, overshoot, and settling time were determined. Observed system responses were similar among the transgenic and virally transduced animals. The CL-FOS and CL-FES systems performed with an average steady state root mean square error (SSRMSE) of 3.01 ± 1.32% and 6.31 ± 2.98%, respectively, during a staircase square wave pattern (reaching 60°, 70°, and 80°). When performing a sinusoidal pattern, qualitatively emulating joint angle oscillations during walking, CL-FOS and CL-FES produced an average SSRMSE of 1.16 ± 0.93% and 11.18 ± 4.32%, respectively (Supplementary Movies 1, 2, 3). In both cases, CL-FOS performed with significantly greater accuracy (unpaired two-tailed *t*-test, *p* < 0.01). Quantification of control metrics (Table 1) for the CL-FOS and CL-FES systems demonstrated that FOS enabled a significantly lower rise time and percent overshoot than FES (unpaired two-tailed *t*-test, *p* < 0.01), given the controller gains tailored to each animal for FOS and FES.

**Table 1:** Control metrics for CL-FES and CL-FOS

	CL-FOS system	*n*	CL-FES system	*n*	*T*-value	*P*-value
Steady-state root mean squared error for square wave pattern	3.01 ± 1.32%	12	6.31 ± 2.98%	11	3.48	0.0022
Steady-state root mean squared error for sinusoidal wave pattern	1.16 ± 0.93%	12	11.18 ± 4.32%	11	7.85	0.0001
Rise time (ms)	29.7 ± 9.4	62	36.91 ± 8.92	47	4.05	0.0001
Percent overshoot by controller	15.6 ± 7.2	40	45.54 ± 1.07	40	26.02	0.0001
*T*_settling_ (ms)	226 ± 140	36	250 ± 39	20	0.78	0.43
*K*_p_	0.08		0.5			
*T*_i_ (s)	0.125		0.0002			

Rise time and percent overshoot by the controller are significantly smaller for FOS when compared to FES as per an unpaired *t*-test at the *p* = 0.01 level. These data were aggregated from trials performed in virally transduced rats

### Mechanism of motor-unit recruitment and muscle fatigue

**Fig. 2:**
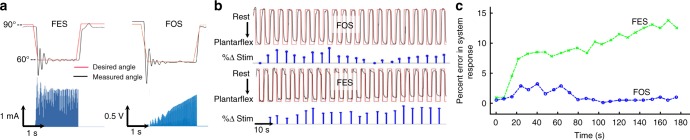
Comparison of recruitment and fatigue between FOS and FES. **a** Comparison of electrical and optical stimulation for CL control. CL-FES uses a more constant stimulation profile as compared to the growing profile of CL-FOS, corresponding to its motor unit recruitment pattern. **b** System response (black) to cyclic CL square wave pattern (red) alternating between rest and plantar flexion (FOS: 1.25 ms PW, 40 Hz, *T*_i_ = 5E−6 s^−1^, *K*_p_ = 1E−2; FES: 100 µs PW, 40 Hz, *T*_i_ = 4E−4 s^−1^, *K*_p_ = 5E−3). Percent change in stimulation (blue) calculated by comparing the peak value during each cycle with the value during the first cycle. **c** Percent error in system response from desired angle for FES and FOS systems over the time course of stimulation. These representative data were drawn from trials performed in rats

The systems’ controllability and stimulation profiles consistently demonstrated that the fiber recruitment mechanism under FOS fundamentally differs from that under FES (Fig. 2a). FOS maintains activation by increasing stimulation amplitude over time to recruit additional fibers. In contrast, FES maintains activation by continued recruitment of the same fibers as indicated by the plateau-shaped stimulation profile. For a 0–30° range of motion, FES utilizes a significantly smaller range of stimulation current, ~60–100% of maximum, to modulate activation as compared to FOS, which utilizes ~20–100%. This was observed in closed-loop tests, whether predominantly driven by proportional gain or integral gain. The larger range of stimulation utilized by FOS may provide more discrete resolution of control, which can be advantageous for complicated control and movement patterns.

We compared the fatigue induced under CL-FES and CL-FOS systems using an extended square wave pattern alternating the joint between rest and plantar flexion (Fig. 2b). This chronic and cyclic pattern was used to mimic movements like walking, which are periodic in nature and occur for extended durations of time. For the CL-FES trial shown in Fig. 2b, the system fatigues at ~20 s and is unable to reach the desired angle, despite increased stimulation, yielding a growing error value (Fig. 2c). Under CL-FOS, we observe an early temporal deviation from the desired angle after activation (at 20 s), but a return to- and maintenance of the desired angle over time (80 s onwards).

### Biophysical mechanism of optogenetic closed-loop control

**Fig. 3:**
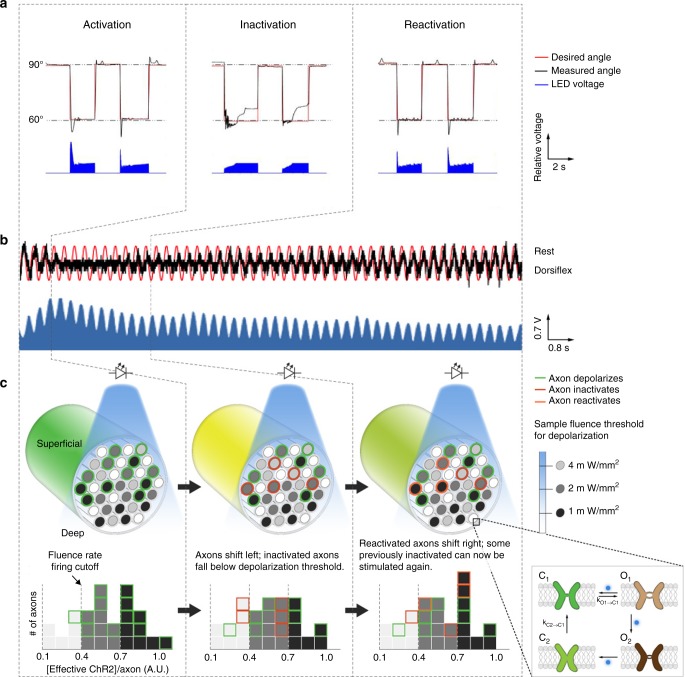
Three-phase, photo-kinetic behavior of optogenetic system. **a** Representative behavior during each phase of the three-phase system response. This representative data are from trials performed in rats. Stimulation is sufficient to maintain the desired position in the activation and reactivation phases, but not the inactivation phase. **b** System response (black) to desired sinusoidal pattern (red) and stimulation delivered by the controller (blue) occurring in the 3 distinct phases of (1) activation, (2) inactivation, and (3) reactivation. This representative data are from a trial performed in a mouse. **c** Schematic depicting optogenetic stimulation of nerve in response to increasing illumination intensities as well as open and closed states modeled for ChR2. [Effective ChR2] is defined as [ChR2_O1_] + [ChR2_O2_] × (*I*_O2_/*I*_O1_). In this representative case, if appropriate dorsiflexion or plantar flexion requires the simultaneous activation of all 12 axons, then it will be achieved in the activation phase but not in the inactivation or reactivation phases. However, if only 9 of the initial 12 axons are required, then angle control will be achieved in the activation and reactivation phases, but not the inactivation phase

We further probed the time variant and nonlinear behavior exhibited by the system under CL-FOS and observed 3 distinct phases in the system response. We provide an example of the system behavior in response to a square wave control signal in each phase in Fig. 3a. In Fig. 3b, we demonstrate the 3-phase behavior during a 200 s span of cyclic sinusoidal movement (alternating the joint between full dorsiflexion and rest) (Supplementary Movie 4) and characterize the phases as follows:

1. Initially, the system achieves the desired target angle.
2. After 10 s of repeated stimulation, the system is unable to achieve the target even under maximal stimulation.
3. Approximately 30 s later, there is an increase in the range of joint angle and at ~100 s, the joint is once again able to reach the desired angle.

We parse this optogenetic motor nerve activation behavior with a 3-phase, photo-kinetic model: (1) activation, (2) inactivation, and (3) reactivation (Fig. 3c). This model builds upon previously identified four-state photocycle kinetics^17,18^, where ChR2 exists in two open conducting states (O1 and O2) and two closed states (dark-adapted = C1 and light-adapted = C2) (Fig. 3c). We define dependencies for transmembrane current and the probability of an action potential. Factors defined in these equations influenced the ability of our system to achieve the control tasks.

When activated by photons, ChR2 switches from C1 to its high-conductance O1 state (C1 → O1), opening a large pore that drives high inward currents via transmembrane cation passage. If illumination ceases while the opsin is in the O1 state, the channel closes back to C1 (O1 → C1). However, if illumination continues, the opsin transitions to O2, (O1 → O2). The O2 state harbors a smaller diameter pore with roughly 20–30% of the conductance of O1 for ChR2^1^ (O1 → O2). If illumination ceases when the opsin is in the O2 state, the opsin closes to C2 and spontaneously converts to C1, through a slower kinetic process. A repeat illumination event while the opsin is in C2 decreases the total change in axonal conductance as compared to that when the opsin is in O1. The resulting current is defined by the following equation

1 $$i_{{\rm transmembrane}} = N_{{\rm Ch},{\rm O}1} \times i_{{\rm O1}} + N_{{\rm Ch},{\rm O}2} \times i_{{\rm O2}},$$

where *i*_transmembrane_ is the transmembrane current, *N*_Ch,O1_, and *N*_Ch,O2_ are the number of channelrhodopsins in the O1 and O2 states, respectively, and *i*_O1_,and *i*_O2_ are the currents transversing each opsin's channel in the O1 and O2 states, respectively.

Assuming conductance of O2 is roughly one-fifth of the conductance of O1[19], we can rearrange the transmembrane current as the following.

2 $$i_{{\rm transmembrane}} = i_{{\rm O1}} \times \left( {N_{{\rm Ch},{\rm O}1} + \frac{{N_{{\rm Ch},{\rm O}2}}}{5}} \right) \\ = i_{{\rm O}1} \times [{\rm Effective}\,{\rm ChR2}]/{\rm axon}.$$

We define the number of opsin molecules in each state multiplied by their relative, normalized transmembrane currents as the ‘Effective ChR2 concentration per axon’. We define the likelihood of eliciting an action potential (AP) as being directly proportional to the cumulative transmembrane current of all opsins.

3 $$P_i\left( {{\rm AP}} \right) = f\left( {N_{{\rm Ch},i},i_{{\rm Ch}},\phi _i} \right) \propto i_{{\rm transmembrane}},$$

where *N*_Ch,*i*_ is the number of opsins in axon *i*, *i*_Ch_ is the current through the opsin channel and *ϕ*_*i*_ is the fluence rate at the axonal surface.

We define the function *g*(*i*) as the contribution of cumulative transmembrane current on the intracellular voltage, and the corresponding intracellular voltage’s effect on the likelihood of reaching a depolarization event. Variables influencing the likelihood of axonal depolarization have been analytically evaluated in previous optogenetic double-cable models^20^. We then express the compound muscle action potential (CMAP) as follows:

4 $${\rm CMAP} = \mathop {\sum }\limits_i^{N_{{\rm ax}}} {\rm MU}_i \times g(i_{{\rm transmembrane}})_i,$$

where MU_*i*_ refers to the voltage generated by each motor-unit and *N*_ax_ refers to the total number of optogenetically active axons in a given nerve.

Axons within a nerve have a range of optogenetic excitability which is dependent on “Effective ChR2”. We bin axons into three categories  of opsin concentration (light gray, dark gray, or black) (Fig 3c). Axons with low opsin concentrations require greater fluence rates to elicit action potentials. Additionally, we bin axons into three categories of activation (green, red, or orange) based on their ability to be depolarized in a given phase (Fig. 3c).

(1) Activation: During the activation phase, optical stimulation drives the system to reach the desired angle consistently. Upon illumination, simultaneous opening of all opsin channels (C1→ O1) results in the highest probability of axonal depolarization due to the large inward current, resulting in high likelihood that nodal transmembrane voltage reaches the −55 mV threshold required to activate voltage-gated sodium channels. Axons in green fire during this state.

(2) Inactivation: In the inactivation phase, while illumination increases to the maximum, the system is unable to maintain the desired angle. During this phase, the majority of the opsins are in the low conductance O2 state and are driven from O2 to the closed C2 state. Together, these dynamics results in a value of ‘Effective ChR2’ that is insufficient for axon depolarization (red axons). The controller consequently increases the illumination intensity in an attempt to activate more compensatory opsins to fire the axon. The remaining axons which establish a superthreshold steady-state between O1 and O2, continue to fire (green axons).

(3) Reactivation: In the reactivation phase, some C2 opsins spontaneously covert to C1 and are activated to the high-conductance O1 state, increasing the 'Effective ChR2' to superthreshold levels (orange axons). As the C2 to C1 transition is independent of optical illumination and is kinetically, a slower process, there is a time lag (~40 s) to re-establish the equilibrium and sufficiently populate C1. Thus, previously subthreshold axons reach the depolarization threshold, enabling the system to reach desired angles at lower levels of illumination.

This 3-phase photo-kinetic process of CL-FOS  is distinct from the temporal dynamics of CL-FES. We can observe this in Fig. 2b which shows the system responses to cyclic square wave control signals. The three phases under CL-FOS are easily contextualized using the error signal (Fig. 2c): (1) With the onset of activation, both systems demonstrate low error. (2) In CL-FOS, inactivation occurs around 20 s, causing an elevation in the error signal. (3) Around 80 s, the CL-FOS error returns to baseline when reactivation occurs. In contrast, the CL-FES system, dominated by fatigue, yields a rising error signal over time with no return to baseline.

We tested the 3-phase photo-kinetic model at varying expression levels and found the activation pattern consistent across expression levels (Supplementary Note 2, Supplementary Figure 5). Additionally, we analyzed the relationship between expression level and the reactivation period. The model suggests a longer period until reactivation for lower expression levels, since fewer excitable opsins (O1, O2) will be available at superthreshold levels during inactivation. Using data from 47 independent trials performed in mice, we found an inverse linear correlation (*r*^2^ = 0.73) (Supplementary Note 3, Supplementary Figure 6) between the expression and reactivation time period.

While the magnitude and time scales varied slightly (as reported in Figs. 2b and 3b), the 3-phase photo-kinetic behavior was observed across square and sinusoidal wave trials, mice and rats, varied expression levels and during stimulation of the tibial and peroneal nerves. Variance in the magnitude and time scale can be attributed to the differences in species, expression levels, number of motor units having expression, and properties of the nerve activated.

We compared the FOS and FES system responses under constant stimulation (40 Hz pulses without rest) and observed an earlier onset of fatigue under FOS in the reactivation phase (Supplementary Note 4, Supplementary Figure 7). This behavior is explained by the 3-phase photo-kinetic model, in which constant stimulation (without dark periods) yields an O1–O2 equilibrium biased toward O2^19^. The O2 state is long-lived and has a low conductance, yielding faster fatigue on the axonal level. As the FOS system employs elevated fluence rates to recruit fibers of lower effective ChR2 to compensate for error, once fatigue begins, the system deviates from the desired angle as the maximum stimulation amplitude is reached. In contrast, under periodic stimulation (Fig. 2b), the periods of darkness during rest enable more inactivated opsins to reset to O1, lowering the activation threshold for the next dorsiflexion/plantar flexion. Thus, compared to FES, FOS demonstrates superior resilience against fatigue in periodic stimulation trials, but inferior resistance against fatigue in constant stimulation trials.

### Direct fascicle state sensing system

For certain neuroprosthetic applications, such as fully implantable closed-loop stimulators, it is desirable to have a direct measurement of fascicle length, as opposed to joint angle or foot position. Therefore, in addition to sensing the distance externally, we utilized sonomicrometry crystals to perform closed-loop control of fascicle length and demonstrate high fidelity tracking.

**Fig. 4:**
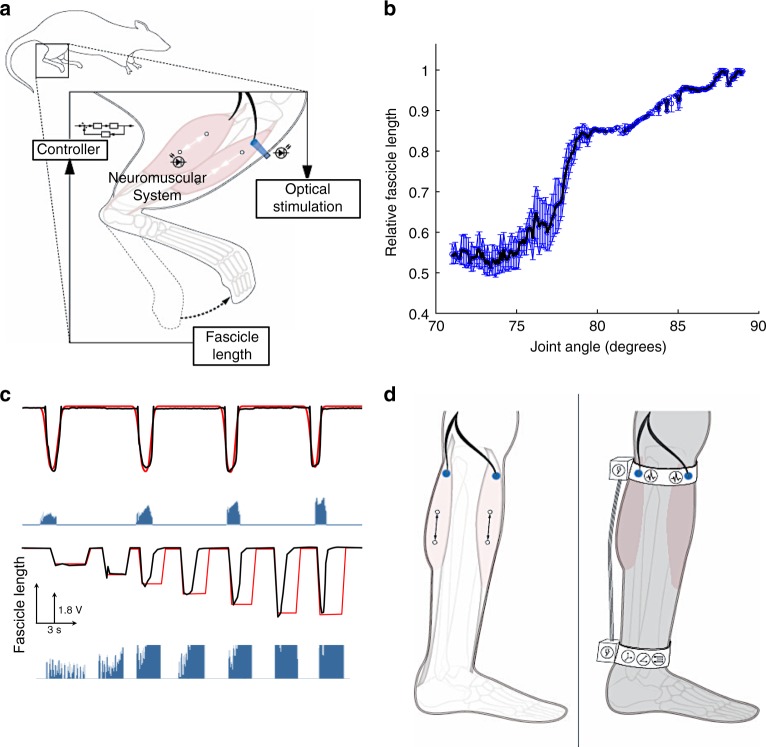
Direct fascicle sensing through sonomicrometry. **a** Schematic depicting closed-loop experimental paradigm using sonomicrometry crystals for fascicle state measurement. **b** Plot of points derived from a closed-loop run defining the fascicle length across the range of joint angles. The relationship between relative fascicle length and joint angle demonstrates passive and active contracting segments throughout range of dorsiflexion (90°–70°). These representative data were obtained from trials in virally transduced rats. **c** Closed-loop control of joint angle using sonomicrometry feedback regarding fascicle length demonstrating smooth sinusoidal movement (top: red—desired, black—actual) and dynamic range in end angle (bottom: red—desired, black—actual). This representative trial was performed in a transgenic mouse. **d** Schematic of the implementation of closed-loop optogenetic/sonomicrometry-driven systems; transdermal (left) and implantable (right)

Sonomicrometry is a sensitive method for fascicle state measurement in murine models and scales well to human implementation^21^. With just two implanted piezoelectric crystals, sonomicrometry uses the time of flight between a transmitted and received pulse, which is linearly related to the physical distance between the crystals. Here, sonomicrometry crystals were sutured to myofibers along the midline of the gastrocnemius and tibialis anterior muscles, without adding tension or disturbing natural anatomy. With sonomicrometry crystals in the loop, instead of the distance sensor, a similar set of trials was performed (Fig. 4a, Supplementary Movie 1). The relationship between joint angle and fascicle length was found to be nonlinear (Fig. 4b), with minimal hysteresis and variance. This non-linearity is due to the active and passive lengthening mechanisms of the muscle fibers under increasing neural activation. However, the lack of significant variability in fascicle length allows the controller to be relatively stable. Using crystal feedback, the system achieved smooth sinusoidal movement and reached various desired angles (Fig. 4c). Throughout these trials, sonomicrometry crystals tracked the fascicle length with sufficient time and spatial resolution to enable closed-loop control.

We observed the aforementioned FOS temporal dynamics in these experiments as well. In Fig. 4c, we observe the opsin switching from its fully active to inactive phase on the third cycle. While the target angle is reached, the system is not able to hold that position due to opsin inactivation, despite saturating levels of stimulation.

Given the adaptability of optogenetic stimulation for both transdermal and implantable implementations, we have provided designs for invasive and non-invasive configurations of a closed-loop optogenetic system for control of the ankle joint (Fig. 4d). In the transdermal approach, LEDs with the appropriate optical wavelengths would be anchored in a band worn in the subpattelar region. LEDs would be positioned over areas of maximum exposure of the nerve to provide optimal light delivery to the peroneal and/or tibial nerves, located within the range of transdermal delivery^22^. A second band worn around the ankle would harbor an inertial measurement unit (IMU). Ankle joint velocity and angle measurements would be transmitted to the on-board microprocessor, which would generate output signals controlling LED illumination. LED-based stimulation would induce neuronal activation, generating muscle contraction, and consequent changes in foot position. These would then be measured by the IMU, closing the loop. In the invasive/implantable approach, sonomicrometry crystals would be implanted along fascicle lines in the appropriate muscles and optical stimulation cuffs would be implanted around the appropriate nerves. Fascicle state measurements from the sonomicrometry crystals would be processed by an implantable microprocessor to generate the output signal for the optical cuff. The optical cuffs would be placed on the peroneal and tibial flexor-extensor pair nerves for activation of the nerves and subsequent control of plantar flexion and dorsiflexion of the ankle joint.

Following all closed-loop testing, nerve tissues were collected from each animal and stained using hematoxylin and eosin to check for any abnormalities or thermal damage induced by stimulation. Histological analysis from all animals demonstrated no abnormal tissue morphology, apoptotic cells, or signs of heat-damage. A representative longitudinal section of tibial nerve stained with hematoxylin and eosin (H&E) from a Thy-1 ChR2 mouse is provided in Supplementary Figure 8.

## Discussion

Electrical stimulation has long been the standard method for neuromuscular stimulation. However, its clinical implementation is riddled with challenges of fatigue, reverse order recruitment of motor units, and diffuse/non-specific excitation of surrounding tissues. Here, we performed CL-FOS and CL-FES of peripheral nerves for position control of the ankle joint to study the behavior, controllability, and practicality of the stimulation methods for motor control implementations in larger-scale systems.

We found that CL-FOS performs with significantly greater accuracy, faster rise times and lesser overshoot when compared to CL-FES. Similar to previous reports^5^, we observed that FOS performs more orderly recruitment of motor units when compared to FES, even in a closed-loop system requiring fast transitions dictated by the control signal. Because of the ability of opsins to reactivate, CL-FOS induces less fatigue when performing periodic movements (as simulated by the sinusoidal and square waveforms), which mimic daily activities such as walking. The temporal dynamics of CL-FOS can be described using a 3-phase photo-kinetic model that is built upon previously described mechanisms of opsin activation^17–20^. Notably, the model suggests that optogenetically active tissues undergo cyclic sensitivity—making them amenable to longer-term, fatigue-resistant stimulation. This starkly contrasts with FES where tissues cannot be reactivated once fatigued.

Given that closed-loop optogenetic stimulation can be done transdermally or through implantable means, we envision closed-loop optogenetic stimulation to aid in a broad range of applications. Recently developed methods to circumvent central motor pathology to restore and control skeletal muscle function have used engrafted cells that can be optically stimulated^23^. Implementing CL-FOS with these grafts would enable therapies for various neurological diseases. Wearable devices incorporating light delivery sources can be worn for conditions such as foot drop or hand tremor to activate or inhibit peripheral nerves and return function to the user. For conditions such as spasticity, muscle rigidity, and tremors, inhibitory opsins would be employed to suppress pathological activation. Alternatively, numerous exoskeleton designs incorporate stimulation to actuate nerves and muscles to work in cooperation with worn hardware^24,25^. These systems currently use FES, but would benefit from the advantages of FOS for fatigue resistance and chronic stimulation. Brain machine interfaces are largely relying on implanted nerve cuffs to provide feedback signals^26^. In addition, recently developed surgical paradigms, such as the agonist–antagonist myoneural interface (AMI), require stimulation of neuromuscular tissues to convey prosthetic feedback to the user about the position and force exerted on prosthetic limbs^27,28^. The ability of the CL-FOS system to perform orderly recruitment would be ideal for these applications. Progress towards these clinical applications requires more thorough study of FOS mechanisms and customization of its properties for each site in the body.

We further compare FES with FOS with regards to practical considerations of (1) position dependence of stimulation, (2) invasiveness and transdermal fidelity, (3) heat-induced damage to tissue, and (4) controllability, which are important for future research and translation.

### Position dependence

Optogenetic stimulation was less dependent on the location of the stimulation because light can transverse through tissues sufficiently to activate small murine nerves. In contrast, electrical stimulation via a stimulating electrode was highly position dependent. Because of the heterogeneous distribution of axons within a nerve, slight shifts in electrode position resulted in vastly different tensions and contacts points between the nerve and electrode. These shifts yielded highly varying activation patterns.

### Specificity and invasiveness

For FES, any break in the insulation of the hook electrode caused activation of surrounding muscles or nerves. In contrast, FOS was performed transdermally with high specificity, activating only the nerve of interest. FOS was performed using LEDs placed on the skin surface in a way that they could be easily removed if necessary. This non-invasive approach was considerably more convenient than FES, which required invasive surgery for direct access of the nerve, laborious manipulation and careful positioning to selectively stimulate the nerve. Transdermal light penetration certainly presents a potential constraint for human translation. Prior studies have shown that a subset of nerves in the human body, including those of interest for upper extremity paralysis control (median and ulnar nerves), are well within the range of successful transdermal optogenetic activation^9^. However, for nerves located deeper within the body, there are numerous studies and research groups working on alternative light delivery mechanisms, implantable hardware (fibers, cuffs), and methods to improve sensitivity of opsins to make them more efficient under transdermal stimulation paradigms^29,30^. Implantable light sources remain advantageous to FOS because they can be implanted at a safe distance from the nerve, precluding the foreign body immune responses and chronic inflammation associated with intrafascicular electrodes and nerve cuffs.

### Heating risks

Damage to tissues through heat dissipated by electrical stimulation and micro-movements of the hook electrode against the nerve have been historical concerns with FES^31,32^. Because FOS requires no direct contact between the stimulating source and the tissue of interest, these concerns are much less severe for FOS.

Heat-induced damage by FOS, caused by heating of the light source, has been previously evaluated^9,33–35^. These studies have yielded guidelines on the optical stimulation parameters which are safe for brain and nerve tissues. The parameters used in this study were well within these safe bounds^9,34,35^. Light delivery and heating of implantable optical devices are valid concerns for translation and a number of strategies and devices, including fiber optics and heat sinks, are being developed to overcome these challenges.

### Controllability

FOS provided a greater degree of controllability and orderly recruitment of motor units when compared to FES, enabling greater opportunity for fine biomimetic control. Orderly recruitment via FOS has been previously attributed to differences in the relevant input resistances of axons or distances between nodes of Ranvier^5,20^. The prevailing models suggest that with a uniform opsin density across all axons, under optical stimulation, smaller motor neurons undergo greater membrane potential changes and thereby depolarize before larger motor neurons. In contrast, under electrical stimulation, larger motor neurons depolarize first due to their lower input resistance for a given applied voltage^5^. We propose the delivery of stimulation to the nerve to potentially contribute to the observed difference in recruitment pattern. For FES, electrical charge is dispersed relatively non-uniformly to the nerve, preferentially targeting  axons adjacent to the contact area of the electrode. In contrast, light homogeneously penetrates through the nerve and has the capacity to recruit motor axons more uniformly. Temporal dynamics of FOS present an important consideration for the development of future control paradigms. The proposed 3-phase photo-kinetic model substantiated the closed-loop system responses observed under a number of conditions in our study. In future system design, these dynamics may be accounted for using adaptive control strategies. By fine tuning the control methods, the cyclic nature of opsin activation may prove advantageous, as it can enable performance over long time periods with minimized fatigue.

Future work should investigate CL-FOS systems utilizing other opsins and biological tissues. Alternate opsins (Halorhodopsins, Chrimson, Chronos, etc.) may offer many of the same benefits of FOS, including decreased fatigue, improved controllability, and orderly recruitment of motor units. However, their photocurrents and expression levels will heavily influence the system properties. Further, the temporal dynamics of activation will be dependent on each individual opsins’ photocycles and kinetics^33,36^. Future work should focus on characterizing the control properties of different classes of opsins to enable a toolbox that empowers researchers to select the optimal dynamics for a given controls application. Additionally, studying FOS systems that modulate other stimulation variables, such as frequency and pulse width, will yield information about the biophysical mechanisms of optogenetic nerve activation. Fatigue-related responses will also be heavily influenced by frequency of stimulation and should be optimized for CL control applications. We posit that, with further development, there is significant potential for FOS to supplant conventional FES systems for research and clinical applications, some of which may extend beyond motor control.

## Methods

### System design

The joint ankle of an animal was driven to desired angles by a control signal via electrical or optical stimulation of the appropriate nerves in the right hind limb. The resulting joint angle was measured (through both invasive and non-invasive methods) and fed into a PI controller. The controller calculated the error between the control signal angle and the measured angle and used this to modulate stimulation intensity, closing the loop.

### Functional optogenetic stimulation system

The ChR2 opsin was chosen to be the model system for its well-characterized properties, fast on/off kinetics^37^, and superior expression profile in the peripheral nerve^33^, which theoretically enabled greater controllability than other opsins. We performed control experiments in both transgenic mice and virally transduced rats expressing the ChR2 opsin to study the system response across a range of animal sizes and take advantage of the more uniform opsin expression available in transgenic animals. Transgenic animals (Thy1-ChR2-YFP) were obtained from Jackson Laboratories and expressed ChR2 fused to YFP under the control of mouse thymus cell antigen (Thy1) promoter. Viral transduction was performed in Fischer 344 rats at 2 days post partum under 2% isoflurane anesthesia with AAV6-hSyn-ChR2 (H134R)-EYFP-WPRE (Virovek). This construct was chosen specifically for its strong photocurrent and favorable expression profile. ChR2 was expressed in the peripheral nerve branches of the sciatic as previously described^9^. Expression was verified using immunofluorescence on harvested tissues, amplifying the YFP signal of the protein complexes. Further histological details are found under the Tissue fixation and histology section.

Stimulation was carried out through 470 nm LEDs (Osram Opto Semiconductors), which were soldered to 26 AWG wires and connected to a T-cube LED current driver (ThorLabs). The maximum current was set to 1.2 A. Custom software (LabVIEW 2017) was created to read the sensed position of the ankle or fascicle state and drive the output voltage via a myRio RT module (National Instruments). The current driver was operated in “Mod” mode, allowing real-time current amplitude modulation proportional to input voltage; the current amplitude ranged from 0 to 1.2 A, which was empirically determined to be linearly scaled to a 0–5 V input. LEDs were placed directly above the peroneal and tibial nerves’ most superficial location to the skin, generally corresponding to the proximal tibia and mid-calf, respectively. Power delivered to the LED can be found by multiplying the current value by the forward voltage of LED, which was 3.2 V for the Osram 470 nm LED used. Illumination power output by the LED ranged from 0 mW to 600 mW, as measured by a digital optical power meter (PM100D, Thorlabs)[9].

A number of pilot experiments, testing a range of frequencies and pulse widths, were performed to determine the modulated variable and stimulation parameters. Frequency modulations led to varying fiber recruitment patterns, which would complicate the ability for us to discern the behavior of the system due to controller dynamics. Additionally, high frequency stimulations would result in inconsistent firing patterns due to refractory kinetics of the opsin^33^. Pulse width modulation was constrained by our hardware and heating concerns. Thus, in this control paradigm, we held frequency and pulse width constant and chose to modulate amplitude. A frequency of 40 Hz was selected as it consistently provided tetanizing contractions in both the rat and mice models, while minimizing refractory inactivation and fatigue. A 5% duty cycle was chosen to limit LED heating from operating at high currents of 1.2 A as well as potential heat-induced tissue damage^9,34,35^. We incorporated safety guidelines about heating and repeated illumination from prior studies^9,33–35^ in the design of our stimulator. During pilot experiments, we recorded EMG from both the agonist and antagonist muscles while stimulating on the nerve. Under both electrical and optogenetic stimulation, we measured no reflex activation in antagonist muscles which may have complicated the controller’s system response. Thus we were able to apply the same controller for the transgenic and virally transduced animals with a parameter optimization protocol described below.

All animal experiments were conducted under the supervision and approval of the Committee on Animal Care at the Massachusetts Institute of Technology.

### Functional electrical stimulation system

To perform electrical stimulation, a custom hook electrode was built using 26 AWG wire connected to a current source (LM234TO-92, Digikey). A schematic of the FES setup is provided in Supplementary Figure 1. Using the 100 Ω resistor, it was empirically determined that an input voltage of 0–1 V corresponded linearly to an output current of 0–10 mA. The stimulation signal was controlled by the myRio to provide 0–10 mA of monophasic current. A pulse width of 100 µs was chosen to balance hardware limits with optimal stimulation under the closed-loop control system (Supplementary Figure 3). The custom LabVIEW software included a variable biasing term to optimize control system performance by accounting for impedance variability in electrode placement and the nerve’s electrical properties. This bias ranged from 0 to 3 mA and was empirically determined for each animal to be the minimum current at which a discernable motor activation (identified either visually or electrically) was produced for each animal.

To expose the nerve for FES, a 1 cm incision was made on the lateral hind limb of adult Fischer 344 rats (8 week) or mice under isoflurane anesthesia using a 15-blade scalpel. Separating the quadriceps muscles from the biceps femoris muscle exposed the sciatic nerve and its branches. The appropriate nerve was lifted from the surrounding tissue using blunt dissection. The custom bipolar hook electrode was placed on the nerve, stabilized using an external device, and insulated using mineral oil (Sigma). A needle electrode (Natus) was placed subcutaneously in the back to serve as a ground.

### Non-invasive closed-loop FOS or FES testing

The right ankle and foot were positioned in a custom-built cradle that loosely suspended the ankle approximately 1.2 cm from the midline. Immobilization of the leg ensured consistent light delivery, isolated movement of the ankle joint and minimal motion artifacts. Clamps lightly immobilized the ankle joint by gently pinning the fat pad between Achilles tendon and tibia, known as Kager’s triangle. This clamp did not penetrate the skin or tendon and did not significantly deform the fat pad so as to affect tendon length or influence the range of motion. The range of motion before and after clamping was physically tested to ensure no restriction of joint movement caused by the clamping. The foot sole was made planar using a thin adhesive card, to prevent errors in distance sensing due to height differences caused by the plantar surface pads of the foot.

Non-invasive external distance sensing was performed using the OADM 12U6460/S35A optical distance sensor (Baumer) driven with a 24 V DC source, whose output was directly read by the custom LabVIEW program. The LabVIEW program converted the distance measurement to the joint angle/position using trigonometric ratios as well as correction factors, which were assigned based on empirical testing.

### Invasive closed-loop FOS or FES testing

For direct fascicle sensing, two uncoated 0.7 mm sonomicrometry crystals (Sonometrics) were sutured intramuscularly and placed approximately 2.5 cm apart in the rat or 1 cm apart in the mouse, inside both the tibialis anterior and gastrocnemius muscles. Fascicle length measurements were used to provide feedback to the control loop in place of the distance sensor measurements.

### Control experiments

All control experiments were performed on both virally transduced rats and transgenic mice. First, using open-loop stimulation, the control system was tuned to adjust to the full range of motion of the joint for each animal. A proportional gain sweep was performed, where the *K*_p_ was increased to explore the entire quasi-stable range. The rise time was the primary metric used to determine the optimum gain. Then, while using the optimal proportional gain, a sweep of the integral time constant was performed. During this sweep, settle time, steady-state error and rise time were balanced to select the most optimal *K*_i_ gain. These parameters were then held constant for subsequent tests and normalized any parameter variation due to varying species, animal size or environmental factors. This process enabled fair comparison of the controller’s performance and system response in control task trials. See Supplementary Note 1.

### Square and sinusoidal movement testing

The system’s controllability was evaluated through control signals with square, sinusoidal, and staircase waveforms. These trials tested the system response while performing dorsiflexion (peroneal nerve stimulation) and plantar flexion (tibial nerve stimulation). Each set of trials consisted of a range of periods, amplitudes, and step sizes of each control signal. Accuracy, rise time, steady-state error and time constants of decay and fatigue were evaluated for each trial. The sinusoidal and square wave patterns mimicked movements of walking and climbing stairs. The staircase control signal tested the ability of the limb to reach discrete angles and maintain that position.

### Muscle fatigue testing

As fatigue is a major concern for nerve stimulation systems, we investigated the fatigue induced by open- and closed-loop FOS and FES systems. Animals were provided with at least 10 min of rest prior to a fatigue trial. To measure the fatigue in a closed-loop paradigm, periodic control signals (square and sinusoidal waves) were delivered for 280 s. In a separate test, we applied constant stimulation in an open-loop fashion for 200 s. The stimulation level was set to drive the position of the ankle to 75% of the maximum. For each trial, the onset and temporal change in system response was monitored.

### Electromyography

To record EMG, four 30G monopolar electromyography (EMG) needles (Natus Medical) were inserted through the skin into the gastrocnemius (GN) and tibialis anterior (TA) muscles for bipolar recording as described previously^9,27^. A monopolar needle electrode (Natus) was placed subcutaneously in the back to serve as a ground. Needles were connected to a 20 kS/s multi-channel amplifier with a fixed 200× gain (IntanTech). Electrical signals controlling the stimulation amplitude, pulse width, and frequency were simultaneously recorded by the amplifier, enabling temporal synchronization of the stimulation and EMG. EMG data were processed in MATLAB.

### Tissue fixation and histology

Rats were killed via intra-peritoneal pentobarbital followed by transcardial perfusion with 60 mL of phosphate buffered saline (PBS) followed by 60 mL of 4% paraformaldehyde (PFA). Sciatic nerves and spinal cords were dissected and fixed for 24 h, paraffin processed, embedded, and cross-sectioned at 10 µm. Sections were stained with hematoxylin and eosin (H&E) and analyzed with an optical light microscope at ×4, ×10, and ×20 magnification to survey for any abnormal morphologies or heat-induced effects.

To verify transduction and quantify expression, immunofluorescence was performed. Expression of ChR2 was amplified with mouse anti-ChR2 at 1:50 (American Research Products, Inc.) and donkey anti-Ms Alexa Fluor 488 (Fisher). Transgenic mice were killed via carbon dioxide inhalation. The sciatic nerve branch was dissected, collected, and fixed in 4% PFA. Tissues were then paraffin processed, embedded, and sectioned longitudinally at 5–10 µm. Expression of the fluorescent reporter, YFP was amplified using Rb pAb anti-GFP (ab290, Abcam) at 1:200. All antibodies were diluted in 1% w/v BSA in PBS-T. DAPI staining was also included to identify cells and perform quantification.

Immunofluorescence images were taken on an Evos FL Auto epifluorescence microscope (Fisher) with identical lighting conditions. Using ImageJ, ChR2 fluorescence per axon was quantified by manually circling individual ChR2 expressing axons, identifying each axon’s average green channel fluorescence on ImageJ, and normalizing between a local non-expressing axon set (minimum intensity) and the global highest expressing axon (maximum intensity).

### Code availability

The code used to run the controller on the LabVIEW platform can be obtained from the corresponding authors upon reasonable request.

## Electronic supplementary material

Supplementary Information

Description of Additional Supplementary Files

Supplementary Movie 1

Supplementary Movie 2

Supplementary Movie 3

Supplementary Movie 4

## Data Availability

The datasets generated during and analyzed during this study are available from the corresponding authors on reasonable request. Code/software used for this study can be requested from the corresponding authors.
