# Supplementary material for: Closed-loop functional optogenetic stimulation
Source: Nat Commun. 2018 Dec 13;9:5303. doi: 10.1038/s41467-018-07721-w (PMC6294002; doi:10.1038/s41467-018-07721-w)
Supplement: Supplementary file 1 — Supplementary Information [file 41467_2018_7721_MOESM1_ESM.pdf]

Supplementary Information

Closed-loop Functional Optogenetic Stimulation

Shriya Srinivasan and Benjamin E. Maimon, Maurizio Diaz, Hyungeun Song, and Hugh M. Herr

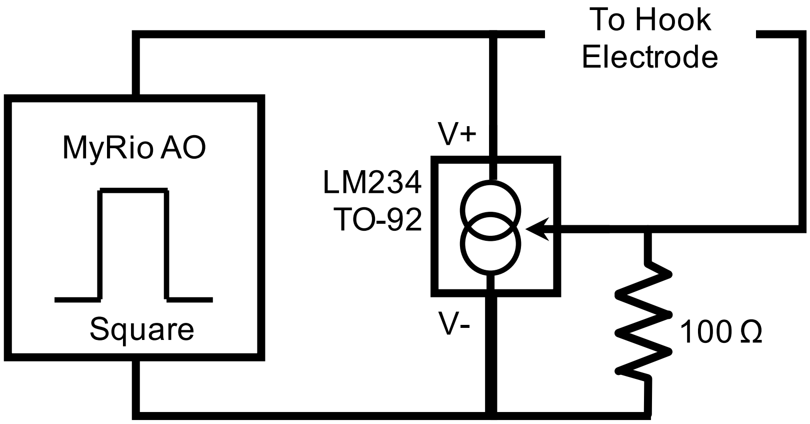

Supplementary Figure 1. Schematic of FES System. This system was used to perform closed-loop fuxnctional electrical stimulation of nerve.

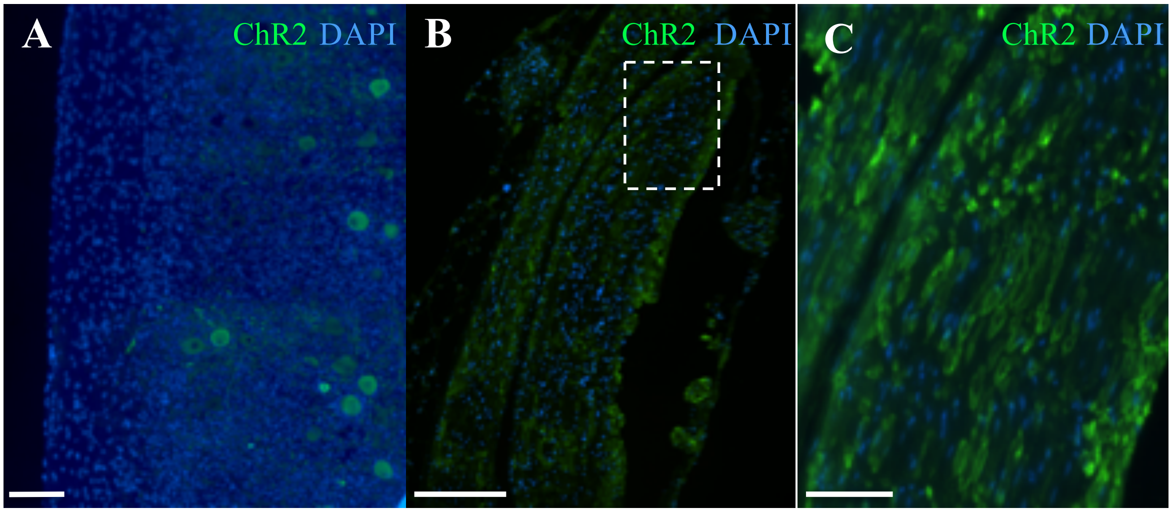

Supplementary Figure 2. Uniform ChR2 Expression in Optogenetically-active Tissues. A) ChR2 expression in the ventral horn motor neurons from lumbar spinal cord sections from virally-transduced rats. Scale bar = 40µm B) ChR2 expression in longitudinally sectioned tibial nerve from transgenic ChR2 mouse. Scale bar = 0.25mm C) Zoomed in section of Supplementary Figure 2B, outlined in the white dotted rectangle, showing ChR2 expression in axons of the tibial nerve from a transgenic ChR2 mouse. Scale bar = 0.06mm.

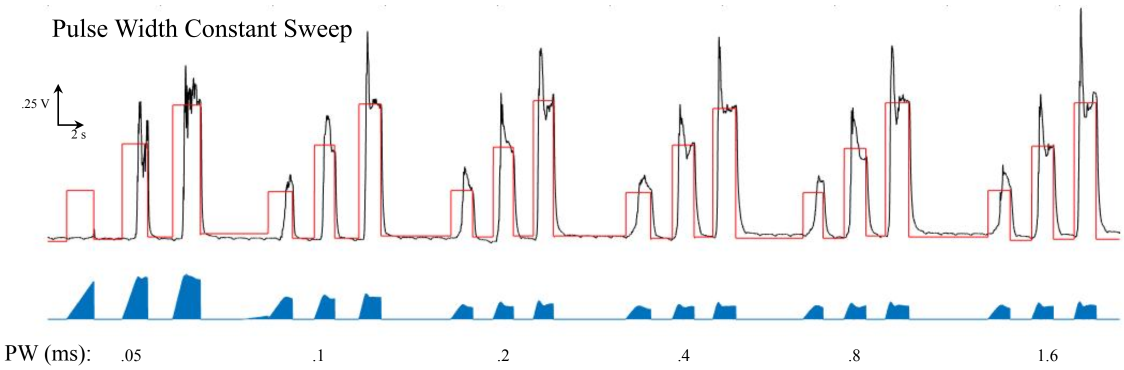

Supplementary Figure 3. **Pulse Width Optimization**. A sweep modulating pulse width of illumination was performed to analyze differences in activation due to FES. Increasing the pulse width decreased the current required for stimulation up to 0.2 ms. From 0.2 ms to 1.6 ms, no significant differences were measured for pulse widths ranging from 0.05 to 1.6ms. These representative data are from trials performed in virally-transduced rats.

Supplementary Note 1

Optimizing System Performance: Gains for the closed-loop system were identified by assessing the stability of the movement, rise time of the system, and overshoot of the system. Integral and proportional gains were swept and the optimal values were chosen for each animal to run the square wave, sinusoidal wave, and cyclic motion pattern control tests (Supplementary Figure 4A).

As opposed to other closed-loop controllers that require only proportional gain, the CL-FOS and CL-FES systems implemented in this study required both proportional and integral components. With only proportional gain, the system was not able to reach and maintain the desired angles, due to its inability to gradually compensate for the time-variant nonlinearities in FOS dynamics – resulting in either no activity or drastic ringing (Supplementary Figure 4C). With predominantly integral gain, the system was able to reach the desired joint angles, but with an elevated rise time (Fig 2B).

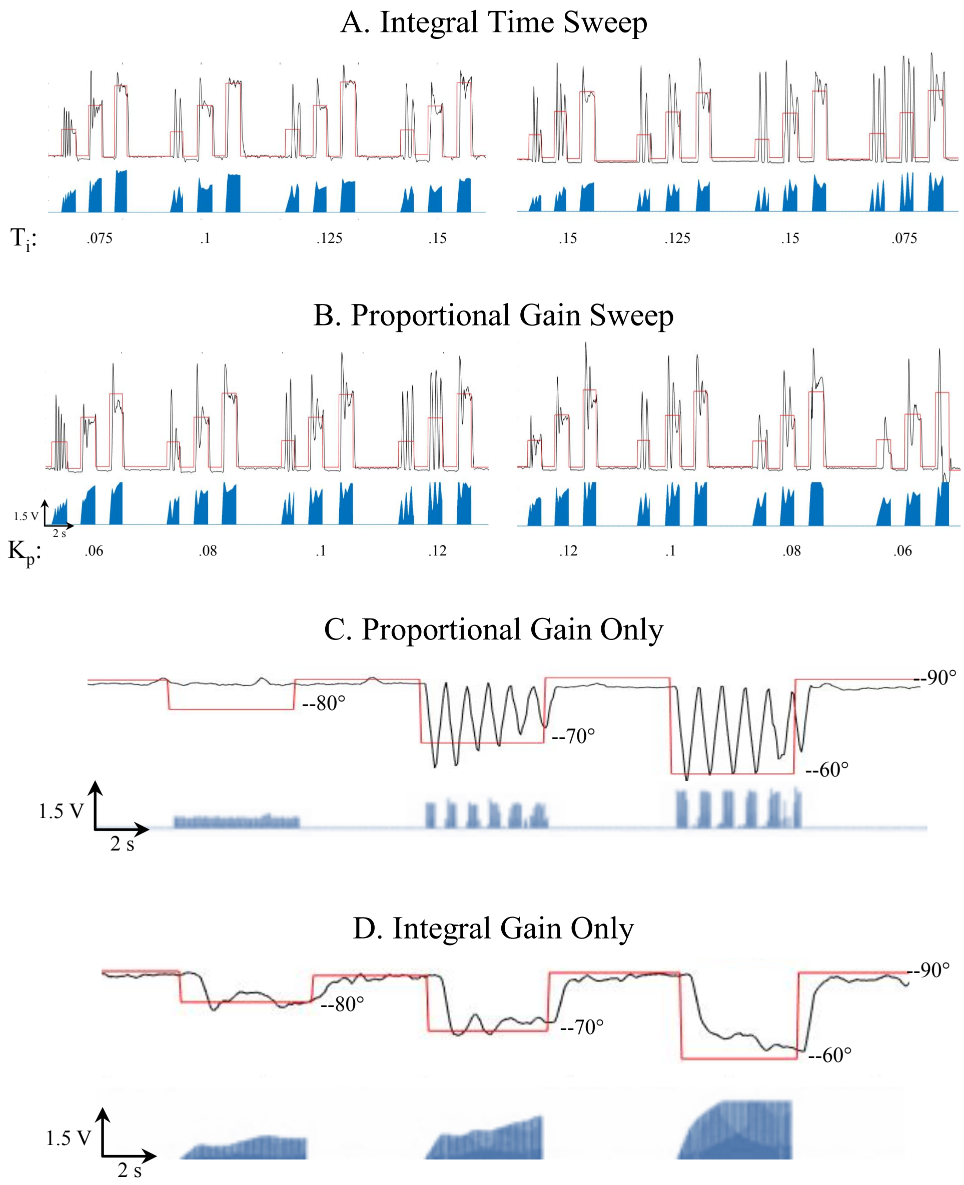

Supplementary Figure 4. CL-FOS System Tuning. Representative data provided from trials performed on a virally-transduced rat. (A, B) Integral and proportional gains were optimized for each animal through a sweep exploring the range of stable movement for the animal (C) System response under only proportional gain is unable to reach and maintain the desired target. Dynamics of opsin activation to the onset of stimulation causes ringing under proportional control only. (D) System response under integral gain is slow to accommodate for error and results in a large rise time.

Supplementary Note 2

FOS Activation is Consistent Across Expression Levels: Naturally, the fluence rate and the level of expression in each animal's nerves has a great influence on the control system's performance. We analyze the expression levels of 5 representative animals from our testing cohort to substantiate assumptions upon which this model stands. For five differentially expressing rats, moving average V_{RMS} EMG in response to illumination of the sciatic nerve was compared to manually counted ChR2+ axon average fluorescence of the peroneal and tibial fascicles of the sciatic nerve, collected immediately following this stimulation trial (Supplementary Figure 3). We identified that rats #1 and #2, which both showed significant counts of axons with high ChR2 (defined as a normalized fluorescence above 0.2) were able to initiate both an initial EMG peak as well as a steady-state EMG during the reactivation phase. Rat #5, which had very few axons with high ChR2, was able to elicit the EMG peak in response to initial illumination but not able to maintain any steady-state EMG during the reactivation phase. Lastly, rats #3 and #4, both of which had very few axons expressing at all, were not able to elicit any EMG during the activation or reactivation periods of the 30 s illumination period. This data suggests that to elicit the sustained, tetanic contractions required for closed-loop optogenetic position control, the presence of many poorly-expressing ChR2+ axons is not sufficient; the presence of axons with high concentrations of ChR2 is required.

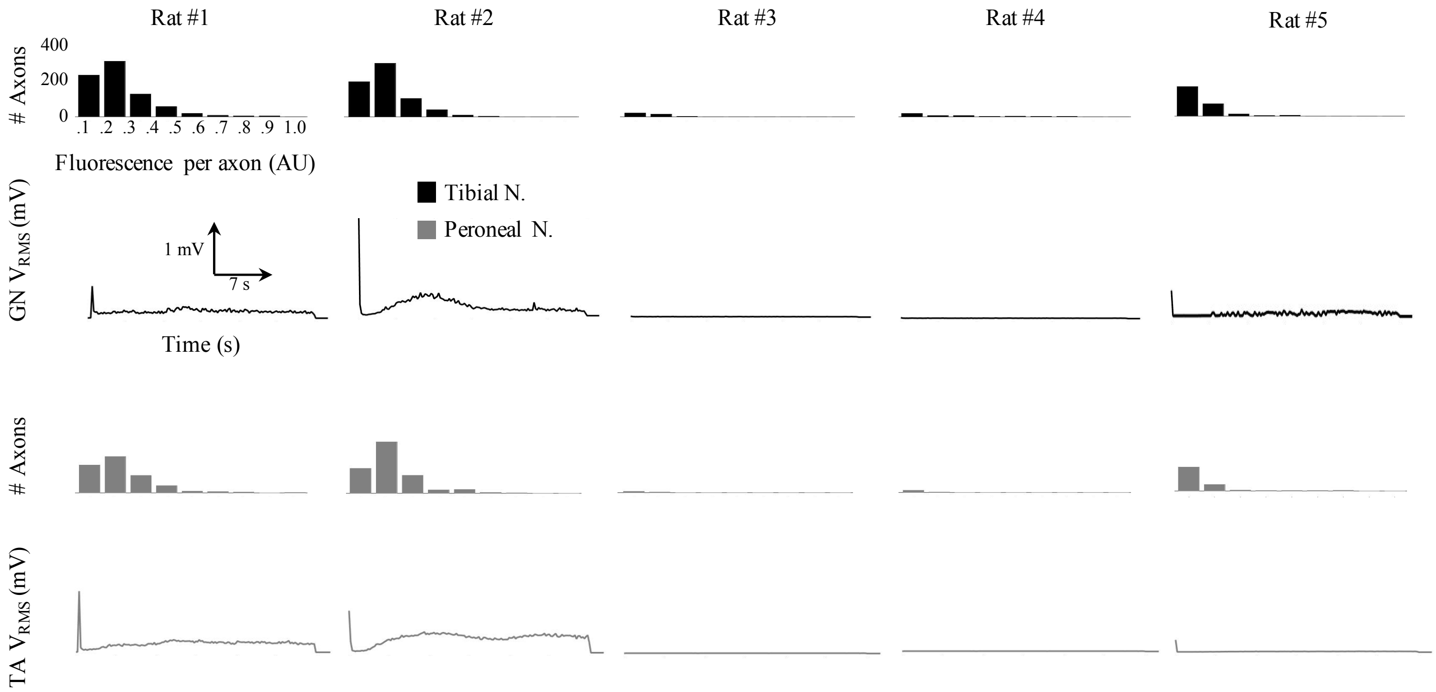

Supplementary Figure 5. Influence of Opsin Expression Level on Neuronal Activation. Comparison of fluorescence per axon histogram and moving average V_{RMS} from 30 s open loop 473 nm illumination of exposed sciatic nerve (45 mW/mm^2, 40 Hz, 10 ms PW) for optogenetically transduced rats at time of euthanasia at 8 weeks post-injection (n=5): GN= Gastrocnemius muscle EMG, TA = Tibialis Anterior muscle EMG. Fluorescence calculated by manually circling individual ChR2+ expressing axons, identifying each axon's average green channel fluorescence on ImageJ, and normalizing to local non-expressing axons (min) and global highest expressing axon (max) with identical lighting conditions.

Supplementary Note 3

Reactivation Time Period is Inversely Correlated to Expression Level: We determine that the overall expression level, defined by a weighted average of the quantity of axons at a given expression level or peak CMAP amplitude[6], moderates the temporal dynamics of the 3-phase behavior. After the onset inactivation, animals with lower overall expression would be expected to require a longer period of time to reach the reactivation phase. This is because there would be fewer axons at superthreshold levels at a given time. Therefore, a greater portion

of opsins in the C1 or C2 state must return to the O1 and O2 states to bring the axon to a superthreshold level. We therefore quantified the average reactivation period, which is equal to the time interval between the EMG peaks of the activation and reactivation phases in 47 animals. We find that there is, as expected, an inverse relationship between expression level and reactivation period (Supplementary Figure 6), which is correlated negatively (slope determining the relationship between peak CMAP amplitude and reactivation period = -0.2774, r^2= 0.73).

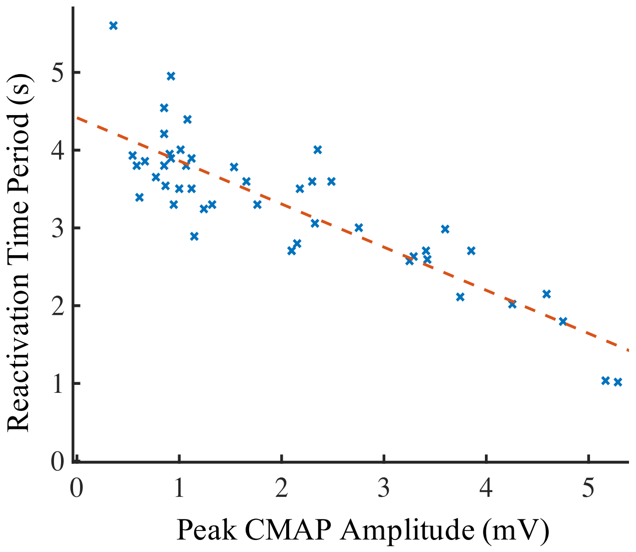

Supplementary Figure 6. Expression Level Influences Reactivation Time. Correlation of the peak CMAP amplitude (representative of expression) to the reactivation time period under constant stimulation for n=47 trials performed in transgenic animals. The reactivation period was defined to be the time interval between the EMG peaks of the activation and inactivation phases. Linear regression yields a slope of = -0.2774 and an r^2= 0.73.

Supplementary Note 4

Mechanism of Recruitment and Muscle Fatigue under Constant Stimulation: We compared the closed-loop behavior of the system under between cyclic FOS patterns and a constant FOS pattern (Supplementary Figure 7).

With constant optogenetic stimulation (40 Hz pulses) the system yielded the 3-phase temporal behavior described in Supplementary Figure 7 above followed by fiber fatigue. We eliminated the potential confounding

variables of electrode placement, pulse width of stimulation and fatigue status through separate experiments. FES yielded maintenance of the desired angle for much longer time periods in both transgenic Thy1-ChR2 mice and virally transduced rats. As explained in the main text, we posit that the difference between CL-FOS and CL-FES occurs due to the photocycle of the ChR2 opsin. To hold a constant angle, the closed-loop system performs continuous stimulation, which causes an O1-O2 equilibrium in the axons that is heavily biased towards O2. In contrast, cyclic movement patterns enable an O1-O2 equilibrium that is much more equally balanced or biased towards O1. Because of the 5-fold higher conductance of O1, the system experiences much less fatigue undergoing cyclic movement than constant movement. We performed a set of these fatigue trials on animals in which no prior testing had been performed to eliminate any potential prior fatigue of the fibers. The results of these experiments were identical to those presented in the main text.

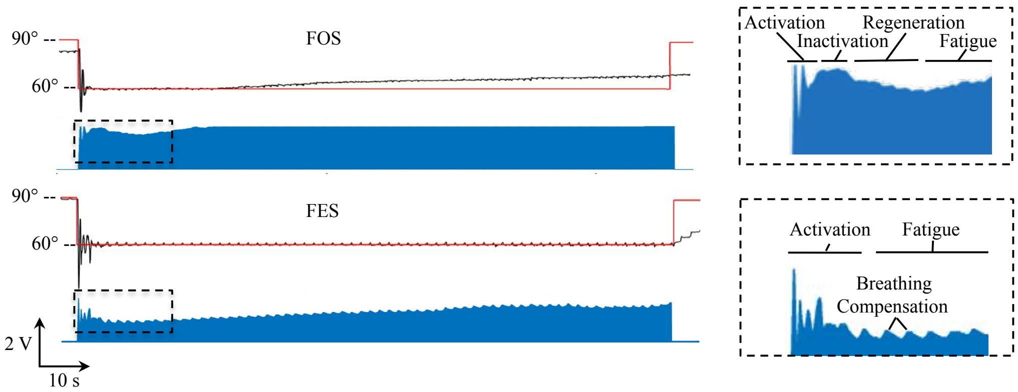

Supplementary Figure 7. System Response to Constant Stimulation. Extended response to closed-loop (100 s stimulation) constant angle pattern for both optical and FES response (optical: 1.25 ms PW, 40 Hz, $T_i = 5E\text{-}6\ s^{-1}$, $K_p = 1E\text{-}2$; FES: 200 µs PW, 40 Hz, $T_i = 4E\text{-}4\ s^{-1}$, $K_p = 5E\text{-}3$). These representative data are from trials performed in virally-transduced rats.

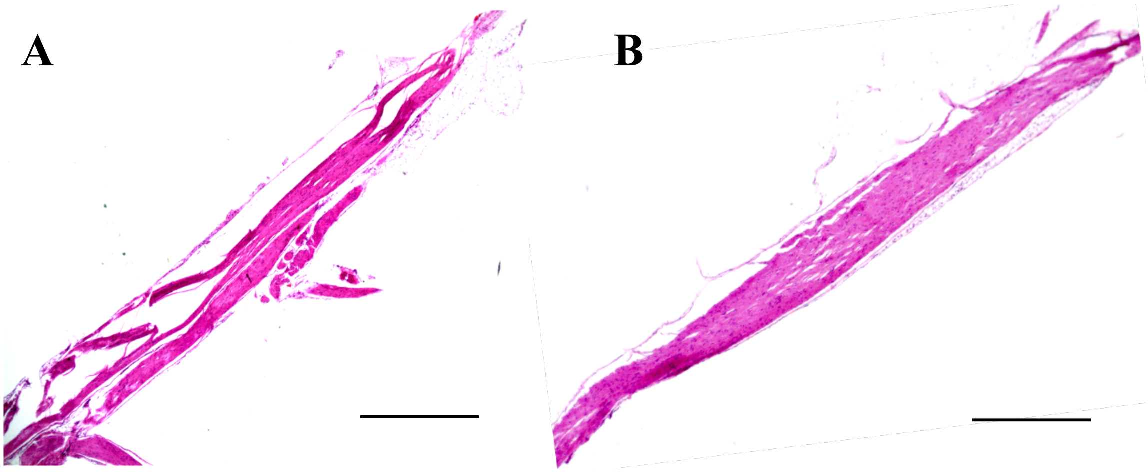

Supplementary Figure 8. Histological Analysis of Heat-induced Daamge. A) Representative H&E stained longitudinal section of tibial nerve from a stimulated transgenic mouse. No abnormal tissue morphology, apoptotic cells, or signs of heat-damage are present. B) Representative H&E stained longitudinal section of tibial nerve from a non-stimulated transgenic mouse demonstrating control morphology. Scale bar represents 1mm.
