# Supplementary material for: Closed-loop functional optogenetic stimulation
Source: Nat Commun. 2018 Dec 13;9:5303. doi: 10.1038/s41467-018-07721-w (PMC6294002; doi:10.1038/s41467-018-07721-w)
Supplement: Supplementary file 2 — Description of Additional Supplementary Files [file 41467_2018_7721_MOESM2_ESM.pdf]

DESCRIPTION OF ADDITIONAL SUPPLEMENTARY FILES

File Name: Supplementary Movie 1
Description: Demonstration of CL-FOS system performing sinusoidal movement task. Video was taken from a trial performed in a transgenic mouse with implanted sonomicrometry crystals, which directly measured fascicle lengths. In addition, an external distance sensor was used (red laser can be seen on the footsole.

File Name: Supplementary Movie 2
Description: CL-FOS system response to staircase square wave control signal. Video was acquired during a trial performed in a virally-transduced rat.

File Name: Supplementary Movie 3
Description: Demonstration of CL-FOS system performing invasive light delivery and response to square wave control signal.

File Name: Supplementary Movie 4
Description: Three-phase photokinetic behavior of CL-FOS system.
